# Extracorporeal Membrane Oxygenation for Acute Respiratory Failure in a Dog †

**DOI:** 10.3390/ani15223247

**Published:** 2025-11-09

**Authors:** Noriko Isayama, Yusuke Uchimura, Kenta Sasaki, Erika Maeda, Toshihisa Takahashi, Megumi Watanabe, Yuji Hamamoto, Takeshi Mizuno, Sayaka Suzuki

**Affiliations:** 1 Uenonomori Animal Hospital, Tokyo 110-0001, Japan; 2 Veterinary Medical Teaching Hospital, Nippon Veterinary and Life Science University, Tokyo 180-8602, Japan; 3 Veterinary Medical Center, Graduate School of Agricultural and Life Sciences, The University of Tokyo, Tokyo 113-0032, Japan; 4 Japan Small Animal Medical Center, Saitama 359-0023, Japan

**Keywords:** extracorporeal membrane oxygenation (ECMO), venoarterial ECMO (VA-ECMO), respiratory failure, cardiopulmonary bypass, dog, case report

## Abstract

A 3-year-old West Highland White Terrier presented to our hospital with dyspnea following aspiration of barium contrast medium during diagnostic imaging for a suspected esophageal foreign body (day 0). Barium contrast radiography had revealed a foreign body in the lower esophagus. During anesthesia, the patient regurgitated and developed respiratory failure and cyanosis. Despite immediate intubation, suction, and ventilatory management, respiratory parameters remained poor. Respiratory support with extracorporeal membrane oxygenation (ECMO) enabled control of blood gas parameters, and tracheobronchial lavage with temporary complete airway occlusion was performed. ECMO was withdrawn once the respiratory status normalized (total support time: 3 h). Considering the possibility of hypoxia-induced brain damage, the patient was extubated on day 1. The dog was alert, changed positions, and drank water independently 5 h after extubation. However, neurological symptoms were observed 1 h later. Cranial magnetic resonance imaging was performed on day 6 owing to persistent neurological symptoms. Although no ECMO-related complications, such as cerebral infarction, hemorrhage, or herniation, were observed, the white matter exhibited hyposignal, indicating hypoxic encephalopathy. The patient died on day 8, without improvement in neurological symptoms. ECMO is an effective treatment option for dogs with respiratory failure, and its prompt introduction may improve survival.

## Simple Summary

Extracorporeal membrane oxygenation (ECMO), a specialized medical technology that temporarily assumes the function of the heart and lungs, allows the body time to recover from severe respiratory failure. Although widely employed in human medicine, its application in veterinary medicine remains limited. Herein, we describe a 3-year-old dog that developed life-threatening respiratory distress after accidentally inhaling barium. Despite intensive ventilatory support, oxygenation failed to improve. ECMO was initiated as a last-resort measure to stabilize respiration, enabling tracheobronchial lavage despite temporary airway occlusion. Following barium removal and 3 h of support, ECMO was discontinued, and the dog regained consciousness. Unfortunately, neurological symptoms later developed, and the dog died 8 days after treatment owing to suspected brain injury caused by prolonged hypoxia before ECMO was initiated. This case highlights the safe application of ECMO in veterinary medicine as a rescue option in severe respiratory failure. It also emphasizes the ethical, logistical, and time-sensitive nature of decision-making in such critical situations. Even when temporary hypoxia occurs owing to diseases, irreversible organ injury may still prevent survival. Establishing clear guidelines and specialized training for veterinary teams is essential to ensure responsible and effective practices, leading to improved outcomes.

## 1. Introduction

Extracorporeal membrane oxygenation (ECMO), a specialized medical technology that temporarily assumes the function of the heart and lungs, is used worldwide in human patients with severe respiratory failure [1,2]. All ECMO systems utilize a pump to remove venous blood from the patient and return it after gas exchange. Venovenous (VV) ECMO returns blood to a vein and is used for gas exchange, whereas venoarterial (VA) ECMO returns blood to an artery and can support circulation. However, ECMO often causes an inflammatory reaction as blood exposed to artificial materials circulates throughout the body [3,4]. This leads to increased vascular permeability and blood coagulation, requiring anticoagulation therapy. Consequently, ECMO is associated with risks of thrombosis, bleeding, infection, and various circulation problems.

Although there are reports of the experimental use of ECMO in dogs [5,6,7] and the use of artificial heart-lung machines for open-heart surgery [8], there are currently no case reports of ECMO use in veterinary medicine, and various aspects of this treatment remain unclear. In the present report, we describe a case of a dog with acute respiratory failure that was successfully introduced to ECMO, managed, weaned, and subsequently extubated.

## 2. Case Presentation

**Figure 1:**
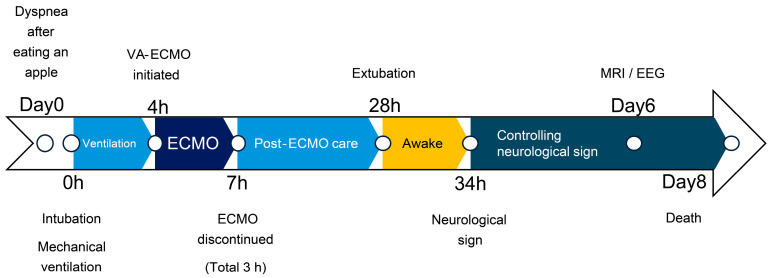
Clinical timeline.

A 3-year-old neutered male West Highland White Terrier (6.5 kg) with no prior medical history presented to the clinic with severe dyspnea after eating an apple (day 0). Barium contrast radiography revealed a foreign body (apple) obstructing the lower esophagus, which was surgically pushed down to the stomach under anesthesia. However, the patient regurgitated and developed respiratory failure and cyanosis, suggesting aspiration. Although immediate intubation and suctioning were performed, the respiratory parameters were showing severe hypoxia and hypercapnia. (Figure 1, Table 1).

One hour following intubation, there was no improvement in respiration. At that point, we proposed either euthanasia or ECMO; however, the owner initially declined, and respiratory support with a ventilator was continued. Various ventilator settings, including high PEEP (>20 cm H_2_O) and high frequency, were attempted, but none improved the respiratory status. These settings were adjusted over time based on SpO_2_ and ETCO_2_ levels. After 2.5 h of ventilation with various settings, severe hypercapnia and hypoxia persisted. Therefore, we concluded that the mechanical ventilation alone could no longer sustain the respiratory status without removing the obstruction. After further consultation with the owner, the dog was planned for tracheobronchial lavage, provided with respiratory support using ECMO.

**Figure 2:**
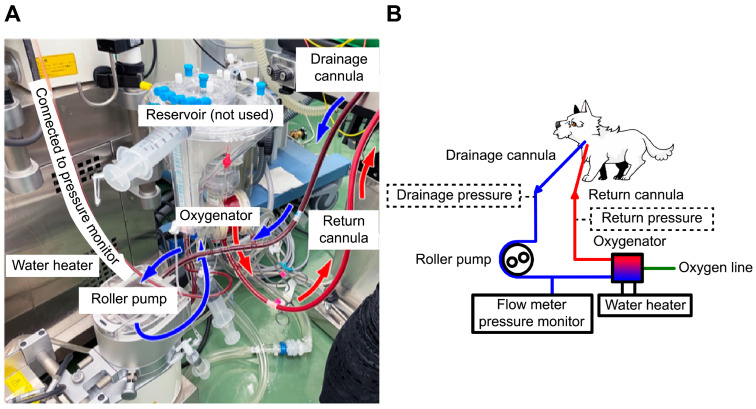
Extracorporeal membrane oxygenation (ECMO) circuit and scheme. (**A**) The ECMO circuit used in this case. A 10-Fr drainage cannula was placed in the left external jugular vein, and a 6-Fr return cannula was placed in the common carotid artery to establish venoarterial ECMO. No reservoir was used because it was substituted with a circuit for cardiopulmonary bypass. (**B**) The circuit scheme for this case. The essential drainage and return pressures, marked with dotted lines, were unavailable in this case. Blue arrow represents venous blood flow, and red arrow represents arterial blood flow.

Heparin (300 U/kg) was administered intravenously, and the measured activated clotting time (ACT) (i-STAT ACT Kaolin; Abbot Japan, Tokyo, Japan) was longer than 250 s. A 10-Fr drainage cannula (Flexmate; Toyobo, Shiga, Japan) was inserted into the left external jugular vein, and a 6-Fr return cannula (DLP Cardiopulmonary Bypass Cannula; Medtronic, Minneapolis, MN, USA) was inserted into the common carotid artery. The dog was connected to an oxygenator and circuit (MERA-HP-Exelung TPC; Senko Medical Instrument, Tokyo, Japan) with a priming volume of 150 mL to establish VA-ECMO (Figure 2).

The MERA-Extracorporeal Circulation System TRUSYS (Senko Medical Instrument) completed the system. The circulatory support flow rate was approximately 40% (250–280 mL/min), maintaining a blood delivery pressure of approximately 120 mmHg with 0.5 L/min of FiO_2_ 1.0. After the ECMO pump was turned on, the respiratory parameters improved. The cannulas were sutured to a drape, which was sutured to the skin to prevent accidental removal. After confirming that the cannulas were secured, tracheobronchial lavage was initiated. Saline (50 mL/kg) was delivered through the tracheal tube to complete airway occlusion with saline (Video S1). After body agitation and saline drainage, barium and a lump of phlegm were removed during the first lavage, resulting in SpO_2_ of 100% and ETCO_2_ of 40 mmHg. White fluid was removed after the second lavage. The patient underwent three lavages in the right and left lateral recumbent positions. Once the drained fluid became clear, SpO_2_ reached 100% and ETCO_2_ decreased to 20 mmHg, allowing the ventilator support setting to be reduced. Suction was used to remove residual saline from the airway, and the dog was maintained in a head-down position. Ventilation was continued with the tracheal tube cuff deflated to allow the airway to dry, After lavage, blood gas analysis revealed marked improvement, and ventilation was gradually reduced.

**Figure 3:**
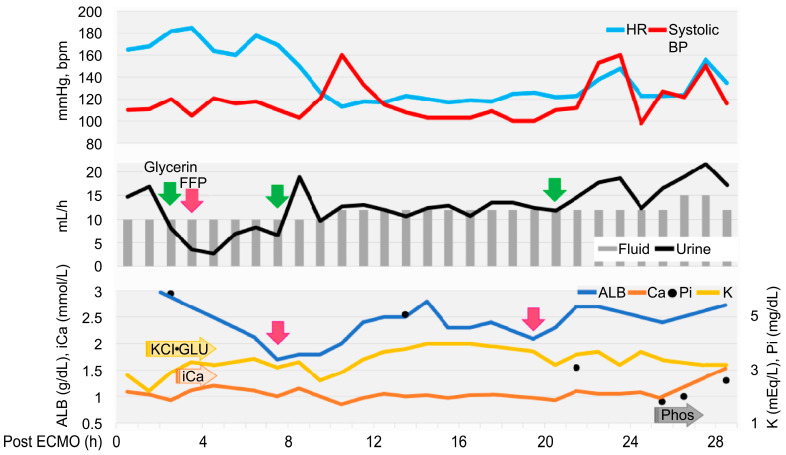
Timeline to extubation after extracorporeal membrane oxygenation withdrawal. Heart rate (HR) and systolic blood pressure (SYS BP) are written on top, fluid volume and urine output in the middle, and albumin (ALB), potassium (K) ion calcium (iCa), and phosphate (iP) below. The reason and timing of glycerol (green) and FFP (pink) administration are indicated by arrows. K, calcium gluconate, glucose, and sodium phosphate were adjusted based on blood test results, approximately every 30–90 min, with continuous infusions started at the arrowed positions.

We continued ECMO support and waited for the respiratory function to recover. The head-down position was discontinued after confirming that no further fluid could be removed through the tracheal tube or suction. ACT was checked every 30 min to maintain >250 s, and 150 IU of heparin was administered once, 90 min after ECMO initiation. The hematocrit level was 43% and the platelet count was 15.2 × 10^4^/μL during ECMO, and no transfusion was required. The patient’s respiratory status remained good, and weaning from ECMO was attempted. The ECMO flow rate was reduced to 8% (10 mL/s), with 0.5 L/min of FiO_2_ 0.3. Weaning was attempted. However, oxygenation worsened. Intravenous furosemide (1 mg/kg) was administered to address suspected acute respiratory distress, possibly related to intubation-associated lung injury, aggressive ventilation, or transient increases in vascular permeability due to ECMO. Subsequently, oxygenation improved, and the patient was weaned successfully from ECMO after a total ECMO support duration of 180 min. Heart rate gradually increased after weaning (Figure 3), and fluid volume was balanced (the delivered fluid volume was approximately equal to the urine volume).

Echocardiography revealed low cardiac volume and tachycardia, suggesting increased vascular permeability and intravascular hypovolemia. Glycerine (10 mL/kg) and fresh frozen plasma (FFP) (15 mL/kg) were administered to prevent cerebral edema. Glucose (1–2 g/kg/h), KCl (0.1–0.3 mEq/kg/h), and calcium gluconate hydrate (10–20 mg/kg/h) were administered continuously and the dose was controlled with blood examination each time. Dobutamine (0.5–3 µg/kg/min) and noradrenaline (0.02–0.1 µg/kg/min) were administered to control blood pressure. Seven hours after ECMO discontinuation, tachycardia improved; however, the albumin level decreased (1.7 g/dL). FFP was re-administered, increasing the albumin level to 2.8 g/dL. Glycerine was also administered to prevent cerebral edema caused by continued low urine output. Urine output increased to 15–30 mL/kg/h 24 h after ECMO discontinuation. Stick urinalysis, performed owing to polyuria, revealed an occult blood reaction (2+) and negative results for glucose, ketones, and bilirubin. The patient was managed under sevoflurane anesthesia and continuous rocuronium bromide infusion (0.4 mg/kg/h) to prevent brain damage due to prolonged hypoxia and high carbon dioxide levels. As the respiratory status was good, sedation was gradually tapered. The patient was switched to spontaneous breathing with pressure support mode. At 26 h after ECMO discontinuation, the results of blood gas analysis, blood biochemistry tests, and complete blood count were satisfactory (hematocrit, 39%; and platelets, 11.5 × 10^4^/μL). The eyelid reflex and swallowing response were promptly observed, and the patient was extubated 28 h after ECMO discontinuation. After recovery from anesthesia, the level of modified Glasgow Coma Scale (MGCS) was 16, and the dog was able to turn around and change its position independently when called by its name. The patient was also able to drink water and consume liquid foods.

**Figure 4:**
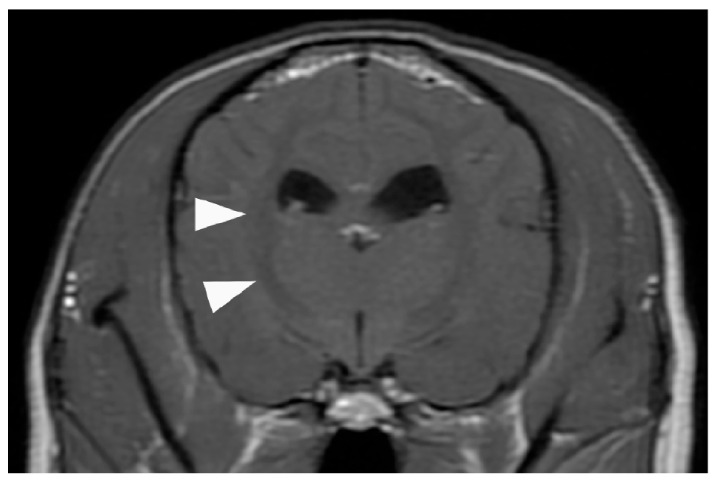
Contrast-enhanced T1-weighted magnetic resonance image of the brain. No common ECMO-related complications suggestive of cerebral herniation due to thrombus, hemorrhage, or edema are observed. However, the white matter exhibits an overall low signal (arrowhead), suspected as brain damage caused by the prolonged hypoxia before ECMO was introduced. ECMO, extracorporeal membrane oxygenation.

However, 6 h after extubation, head tremors were observed (Video S2). Temporary improvement was observed after intravenous administration of diazepam (0.5 mg/kg). However, as the tremors continued, continuous diazepam was administered. Hyperthermia, panting, and involuntary movements developed, and the MGCS dropped to 10. Sedation was reinforced, but repeated panting and respiratory arrest occurred; therefore, the patient was intubated again 18 h after extubation, and respiratory control was performed. Given the pronounced neurological symptoms, we considered the high body temperature and panting to be attributed to brain damage and repeated administration of glycerine and anticonvulsants. The patient was extubated after the respiratory arrest resolved, although sedation was continued. However, the high body temperature and involuntary movements persisted. On day 6, the patient’s hyperthermia and involuntary movements improved, and all medications used for sedation management were discontinued. However, the patient’s consciousness did not improve, although mild eyelid reflex and swallowing response were observed. Cranial magnetic resonance imaging (MRI) and electroencephalography (EEG) were performed to assess brain damage, revealing that the white matter exhibited hyposignal on T1-weighted and post-contrast T1-weighted MRI (Figure 4).

No hemorrhage, infarction, cerebral edema, or cerebral herniation was observed. EEG examination revealed no apparent abnormal waveforms. The eyelid reflex and swallowing response progressively weakened, the MGCS was reduced to 3, and the patient died on day 8 from respiratory arrest without regaining consciousness. The owner did not request further resuscitation.

## 3. Discussion

According to the Extracorporeal Life Support Organization guidelines, ECMO is indicated for bronchoalveolar lavage in cases of severe inhalation injury. In the present case, the patient developed upper airway obstruction secondary to barium aspiration. This represented a severe inhalation injury meeting the criteria for extensive bronchoalveolar lavage, as defined by [9], the International Organization for Human ECMO. Given that the patient was young (3 years old), exhibited good energy and appetite, and had no underlying chronic diseases, a full recovery to a normal life was expected. Therefore, ECMO was not contraindicated and was indicated for prompt introduction. However, owing to the reluctance of the owner and our inexperience in using the modality, a considerable delay occurred between the presentation with respiratory failure and the introduction of ECMO, resulting in prolonged hypoxia. MRI later confirmed hypoxic–ischemic brain injury (HIBI), and with no other reversible causes identified, hypoxia was deemed the most likely cause of death. HIBI is recognized as a potential neurological complication following ECMO [10] and may also be associated with the pre-ECMO status of the patient [11]. However, determining whether HIBI results primarily from the pre-ECMO status or from the ECMO procedure itself remains difficult [11,12]. Both pre-existing hypoxia and ECMO-related factors may interactively contribute to the development of HIBI. Although research in this field proves challenging, further studies are needed to clarify the mechanisms underlying HIBI in veterinary ECMO cases.

In the present report, during ECMO support, oxygenation was maintained, which enabled tracheobronchial lavage with complete airway occlusion. Following the lavage procedure performed under ECMO support, barium was completely removed, and the respiratory status drastically improved; the dog was extubated and allowed to drink water. MRI demonstrated no brain herniation or ECMO-related complications commonly reported in human medicine, such as cerebral hemorrhage or stroke [4,13]. Therefore, early ECMO intervention likely could have prevented hypoxia-related brain damage and improved survival [12]. Currently, there are no established criteria for ECMO use in veterinary medicine. However, human studies have indicated that early initiation of ECMO can improve survival in cases of acute respiratory distress syndrome [14], pulmonary hypertension [15], coronavirus disease 2019 [16], and cardiogenic shock [17,18,19]. These findings suggest that the same principle may apply in veterinary medicine, emphasizing that ECMO should be initiated proactively and without delay when a high probability of survival is expected.

In this case, the ECMO pump flow rate was only approximately 50% of the adult dose and 40% of the pediatric dose recommended by the guidelines [20,21,22]. The cardiopulmonary bypass circuit used for ECMO lacked a pressure monitoring line, as it was adapted from the circuit routinely used for canine open-heart surgery at our facility. Consequently, the pressure monitor was only positioned before the oxygenator, and the ECMO was operated based on the oxygenator pressure as the primary indicator. As prolonged ECMO use may increase the risk of thrombosis and circuit-related complications, both drainage and return pressures should be monitored to enable precise flow rate adjustment (Figure 2B) [20]. However, in this case, the absence of drainage pressure monitoring limited our ability to increase the pump flow rate.

Immediately after ECMO initiation, the SpO_2_ increased from 63% to 90%; however, the SaO_2_ did not improve. This discrepancy was likely attributed to the use of VA-ECMO and the common carotid artery for the return cannula. While VV-ECMO mixes oxygenated blood in the veins, VA-ECMO mixes oxygenated blood in the arteries. In this case, the mixing point was presumed to be near the brachiocephalic artery, as the blood was pumped at a low flow rate into the left common carotid artery [9]. Therefore, the oxygenated blood to the head improved the tongue SpO_2_ reading. However, as the pump flow rate was approximately 40% and the blood was collected from the hind leg, an increase in SaO_2_ could not be confirmed. Owing to the small body size, a 10 Fr drainage cannula was placed in the jugular vein. Moreover, performing VV-ECMO would have required an abdominal venous approach, which carries additional surgical and hemodynamic risks. The main objective of ECMO in this case was to complete tracheobronchial lavage, which involved moving the dog. To minimize the risk of catheter dislodgement, VA-ECMO was selected, as it could be performed entirely through a cervical incision and allowed for rapid cannulation. Moreover, as VV-ECMO in dogs remains largely experimental with no established configuration, VA-ECMO was selected because it could be performed using a cardiopulmonary bypass system identical to that used for routine open-heart surgery at our hospital. This approach allowed our surgical team to ensure safety and precise flow control. However, future studies involving canine subjects are warranted to compare the flow dynamics and gas exchange efficiency between VA-ECMO and VV-ECMO. Such comparative research will be essential to determine the optimal ECMO configuration for different clinical objectives and patient sizes in the veterinary field.

In human medicine, ECMO use is associated with ECMO- and patient-related complications [1,23]. Our case exhibited no ECMO-related complications, such as thrombosis, bleeding, or problems with the oxygenator, presumably because the total ECMO use was only 3 h, limiting the opportunities for such events to occur. During airway lavage, the cannula or circuit may cause issues; however, careful fixation of the cannula likely prevented accidental dislodgement. We also did not observe patient-related complications, such as bleeding, hemolysis, or infection, possibly owing to the short duration of ECMO support; however, a urine test revealed an occult blood reaction of 2+. In human ECMO, anemia often progresses owing to blood consumption by the pump, even in the absence of visible hemolysis [24]. Therefore, red blood cell transfusion might be necessary in prolonged cases. Patient-related complications also include decreased circulating blood volume and altered hemodynamics, largely due to increased total body water volume from the ECMO circuit and increased vascular permeability secondary to foreign-body reactions. These mechanisms can lead to generalized edema, including pulmonary edema, and cerebral herniation [13,25]. In our case, hypoalbuminemia secondary to intravascular hypovolemia and increased vascular permeability occurred within 24 h after weaning from ECMO, accompanied by unstable circulatory dynamics within 8 h after weaning. The total FFP volume used in this case was 110 mL. In human medicine, blood transfusion is routinely required when ECMO is applied to patients weighing less than 10–15 kg [20]. However, in our case, the blood transfusion volume per body weight was higher than that typically reported in humans [24], possibly reflecting a higher foreign-body exposure per body surface area in this dog than in humans, as well as species-related differences. Although the dog weighed 6.5 kg and the hemodilution rate was low, smaller patients may require a red blood cell transfusion. Complications similar to those observed in human medicine were observed in our case, despite the relatively short duration of ECMO. Notably, the severity and timing may vary depending on the support duration and patient size. Therefore, further studies are required to determine appropriate management strategies for dogs during and after ECMO support.

Currently, no standardized criteria for ECMO exist in veterinary medicine. In this case, ECMO was introduced as a last-resort oxygenation support after all conventional respiratory interventions failed and was used briefly during tracheobronchial lavage and recovery. In veterinary practice, ECMO poses logistical and ethical challenges; its initiation must balance potential survival benefits against financial and institutional feasibility. Although this case was successfully weaned, ethical dilemmas may arise when discontinuation is uncertain. Recent human data report 30-day survival rates of approximately 52% for VV-ECOM and 34% for VA-ECMO, highlighting the importance of owner consent and appropriate case selection [26,27]. Applicable cases may vary widely depending on the method of ECMO implementation in animals, management of ECMO-related complications, and patient care before and after the introduction of ECMO. While prognostic tools exist in human ECMO, no equivalent framework currently exists for veterinary applications [28]. Overall, ECMO is expected to become an effective treatment option in veterinary medicine once management methods improve and applicable cases are identified. However, ECMO is only a time-saving device to protect organs from hypoxia and provide time for the treatment of the underlying disease.

## 4. Conclusions

This case demonstrates that ECMO can be safely applied to dogs as a rescue option in severe respiratory failure, suggesting its technical feasibility in veterinary settings. However, further studies are needed to clarify the indications, management, and evaluation of patients before, during, and after treatment in veterinary medicine. This case also highlights the ethical and time-sensitive nature of decision-making in such critical situations. Even when hypoxia occurs temporarily as a result of disease, irreversible organ injury may prevent survival despite successful treatment of the primary disease. Overall, ECMO has promising potential in veterinary medicine, and prompt decision-making and timely implementation are essential to maximize its benefits.

## Figures and Tables

**Table 1:** Blood gases, SPO_2_, ETCO_2_, and ventilator settings at different time points.

	Time (hour)	Intubate	0.5 h	1 h	1.5 h	2.5 h	4 h Start ECMO	5 h Post Lavage	6 h Beginning of Weaning	7 h Wean Off ECMO	24 h from ECMO	26 h from ECMO
pH		7.003	6.790	7.121	7.043	7.073	7.236	7.488	7.357	7.344	7.305	7.338
PCO_2_		120.0	130.0	91.0	122.6	118.5	70.3	23.1	30.1	43.7	51	47
PO_2_		61	85	44	46	46	43	112	75	215	187	126
SPO_2_		77	88	70	79	80	90	100	88	99	100	100
ETCO_2_		670	390	600	810	840	450	200	300	390	54	37
P/F		61	85	44	46	46	43	186	107	305	311	315
RR		60	30	40	50	50	40	10	30	25	32	20
PEEP			2	5–10	6–20<	6–15	6	6	6	6	6	6
PIP		20	25	25–35	35–45	35–45	36	26	26	26	20	15

PaCO_2_, partial pressure of carbon dioxide in arterial blood; PaO_2_, partial pressure of oxygen in arterial blood; SpO_2_, peripheral oxygen saturation; ETCO_2_, end-tidal carbon dioxide; P/F ratio, the ratio of arterial oxygen partial pressure (PaO_2_) to fractional inspired oxygen (FiO_2_); ECMO, extracorporeal membrane oxygenation. After the start of ECMO, SPO_2_, ETCO_2_, pH, and CO_2_ improved dramatically, but O_2_ did not (4 h). After airway lavage, all respiratory parameters improved.

## Data Availability

The original contributions presented in the study are included in the article/Supplementary Material. Further inquiries can be directed to the corresponding author.

## Supplementary Materials

The following supporting information can be downloaded at: https://www.mdpi.com/article/10.3390/ani15223247/s1; Video S1: Video of the tracheobronchial lavage; Video S2: Video of the head tremor.

## Abbreviations

The following abbreviations are used in this manuscript:

ECMO	Extracorporeal membrane oxygenation
EEG	Electroencephalography
ETCO_2_	End-tidal carbon dioxide
FiO_2_	Fraction of inspired oxygen
FFP	Fresh frozen plasma
HIBI	Hypoxic–ischemic brain injury
MGCS	Modified Glasgow Coma Scale
MRI	Magnetic resonance imaging
pCO_2_	Partial pressure of carbon dioxide
PEEP	Positive end-expiratory pressure
PIP	Peak inspiratory pressure
pO_2_	Partial pressure of oxygen
RR	Respiration rate
SpO_2_	Oxygen saturation
VA-ECMO	Venoarterial extracorporeal membrane oxygenation
VV-ECMO	Venovenous extracorporeal membrane oxygenation
